# Synthesis of Cellulose Acetate Butyrate Microspheres as Precursor for Hard Carbon-Based Electrodes in Symmetric Supercapacitors

**DOI:** 10.3390/polym16152176

**Published:** 2024-07-30

**Authors:** Johanna Fischer, Katrin Thümmler, Igor Zlotnikov, Daria Mikhailova, Steffen Fischer

**Affiliations:** 1 Institute of Plant and Wood Chemistry, TUD Dresden University of Technology, Pienner Str. 19, 01737 Tharandt, Germany; katrin.thuemmler@tu-dresden.de; 2 Leibniz Institute for Solid State and Material Research (IFW) Dresden e.V., Institute for Materials Chemistry (IMC), Helmholtzstraße 20, 01069 Dresden, Germany; d.mikhailova@ifw-dresden.de; 3 B CUBE—Center for Molecular Bioengineering, TUD Dresden University of Technology, Tatzberg 41, 01307 Dresden, Germany; igor.zlotnikov@tu-dresden.de

**Keywords:** cellulose microspheres, cellulose acetate butyrate, activated carbons, electrochemical energy storage

## Abstract

Cellulose microspheres have a wide range of applications due to their unique properties and versatility. Various preparation methods have been explored to tailor these microspheres for specific applications. Among these methods, the acetate method using cellulose acetate is well known. However, replacement of the acetate group through the butyrate group significantly extends the variety of morphological properties. In the present work, microspheres based on cellulose acetate butyrate are being developed with modified characteristics in terms of particle size, porosity, surface morphology and the inner structure of the microspheres. While the inner structure of cellulose acetate microspheres is predominantly porous, microspheres prepared from cellulose acetate butyrate are mainly filled or contain several smaller microspheres. Carbon materials from cellulose acetate butyrate microspheres exhibit a high specific surface area of 567 m^2^ g^−1^, even without further activation. Activation processes can further increase the specific surface area, accompanied by an adaptation of the pore structure. The prepared carbons show promising results in symmetrical supercapacitors with aqueous 6 M KOH electrolytes. Activated carbons derived from cellulose acetate butyrate microspheres demonstrate an energy density of 12 Wh kg^−1^ at a power density of 0.9 kW kg^−1^.

## 1. Introduction

For the preparation of cellulose-based microspheres, a variety of preparation methods are known, resulting in a wide range of particle sizes from 1 µm to 3 mm. In this work, the acetate method [1,2] is used to obtain homogeneous particles with a particle size less than 5 µm and a very narrow particle size distribution. The cellulose microspheres have high and controllable porosity in combination with high mechanical strength and chemical reactivity. Further, they possess a good hydrophilicity and are insoluble in most organic solvents. The many possibilities for functionalisation allow an optimal adaption of the spherical particles to the respective application [3,4,5].

Cellulose microspheres can be used in a wide range of applications, from pharmacy to chromatography and water purification. In the pharmaceutical sector, the beads function as adsorbents in extracorporeal blood purification or for the controlled release of active ingredients [3,6,7,8,9]. For the purification of water, functionalised beads are used to adsorb metals from aqueous solutions [3,4,7,8]. Commercially available cellulose microspheres such as Cellufine^®^ with a particle size of 40–130 µm, or Cytopore™ with a particle size of 200–280 µm, are used for chromatography or in suspension culture systems to support cell growth, respectively [10,11].

**Figure 1:**
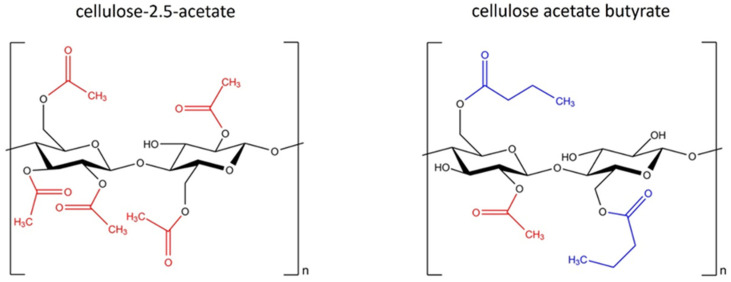
Structural formula of cellulose-2.5-acetate and cellulose acetate butyrate.

The two cellulose esters used in this work, cellulose-2.5-acetate and cellulose acetate butyrate, differ primarily in terms of their substituents (Figure 1). The properties of cellulose esters are significantly influenced by the type of substituent, the degree of substitution and the distribution of the functional groups along the chain [12].

Cellulose acetate butyrate (CAB) is currently used mainly either in the pharmaceutical sector for the encapsulation of active ingredients such as repaglinide or ketoprofen, or as a coating material. CAB is the most commonly used cellulose ester for encapsulation due to its high UV stability, good film forming properties and good tolerability. They are usually prepared by the emulsion–solvent evaporation method, which results in particle sizes in the range of 100 to 600 µm, depending on the active ingredient contained. Another method to obtain more homogeneous and smaller particles < 10 µm is spray drying [13,14,15,16].

In this work, microspheres with cellulose acetate butyrate or a mixture of cellulose acetate and cellulose acetate butyrate using the acetate method are prepared in order to obtain relatively homogeneous microspheres with a particle size < 5 µm. We have investigated how the physical characteristics as morphology, particle size, porosity and thermal behaviour can be varied with the use of cellulose acetate butyrate. In addition, the first orientation studies on the inner structure of the microspheres are performed.

Electrochemical energy storage systems are very important for the reliable availability of electrical energy. In the past years, electrochemical capacitors, also known as supercapacitors, have come into research focus. Supercapacitors offer extremely high power densities combined with short charging and discharging processes in the range of seconds, and a long cycle life [17,18,19]. Due to their high power density and the fast available energy, supercapacitors are mainly used for uninterruptible power supplies as well as in fire and emergency systems or for regenerative braking [18,20]. Supercapacitors can be divided into two categories, electrical double layer capacitors (EDLC) and pseudocapacitors, which differ in the type of energy storage mechanisms. The choice of the electrode material depends mainly on the type of storage mechanism. For the EDLCs, activated carbons with a high specific surface area are the most commonly used electrode materials. A variety of carbonaceous precursor materials can be used to prepare activated carbons. Thereby, the demand for more sustainable materials is constantly increasing [19,21]. Also, carbon doping with various heteroatoms such as phosphorous, nitrogen or oxygen is investigated to improve the electrochemical performance through functional groups on the surface of carbons [22].

Our previous works have shown that carbons derived from cellulose acetate microspheres are promising as electrode materials in symmetrical supercapacitors with aqueous electrolytes [23,24] and as anode materials in Li-Ion batteries [25]. In this work, carbons from the prepared cellulose acetate butyrate microspheres were also tested as electrode materials in supercapacitors. It is assumed that the advantages over cellulose acetate microspheres are due to the modified surface functionality, the higher carbon amount and the changed morphology of the microspheres. The relationship between the properties and the structure of the microspheres and the properties of the resulting carbons was investigated. 

## 2. Materials and Methods

### 2.1. Preparation of Cellulose Acetate Butyrate Microspheres

**Figure 2:**
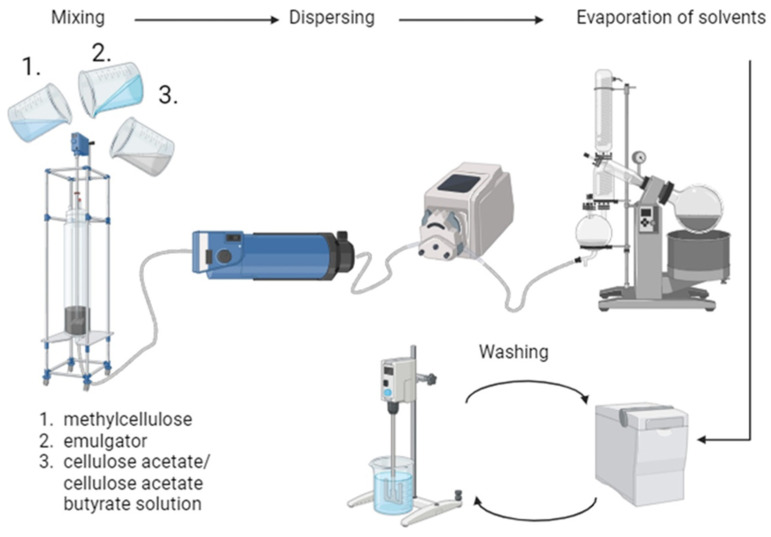
Schematic illustration of the preparation process of cellulose acetate (butyrate) microspheres. Created with BioRender.com.

First, solutions of cellulose acetate and cellulose acetate butyrate as well as methylcellulose (Methocel, viscosity 1200–1800 mPas) as protective colloid were prepared in order to produce microspheres by the acetate process [1]. Both cellulose-2.5-acetate (Aceplast, Acetati SpA, Verbania, Italy) and cellulose acetate butyrate (Sigma Aldrich, St. Louis, MO, USA, 180963) were dissolved in ethyl acetate and methanol under stirring, while methylcellulose was dissolved in water. For the preparation, different ratios of cellulose acetate (CA) and cellulose acetate butyrate (CAB) solutions were mixed. The microspheres are labelled with the amount of CAB used, e.g., the microspheres made entirely of CAB are named CAB100, while the mixed product of 25% CA and 75% CAB solution is named as CAB75. The dissolved methylcellulose was mixed with salt (sodium acetate trihydrate, Roth) and surfactant (Triton X-100, Sigma Aldrich), and afterwards the CA/CAB solution was added. In order to form microspheres, the emulsion was dispersed by an inline Turrax, and the solvents were evaporated. This process, schematically depicted in Figure 2, is a further development of the acetate process elaborated by Thümmler et al. [2]. The samples were washed several times by centrifugation and resuspension in water. For further characterisation and carbonisation steps the samples were freeze-dried. 

### 2.2. Carbonisation and Activation

The prepared microspheres were carbonised for 2 h at 800 °C with a heating rate of 300 K h^−1^ in an argon atmosphere (argon flow with 0.2 L min^−1^). For CAB100, an activation step with potassium hydroxide was performed. The precursor material was mixed with a KOH solution with a cellulose/KOH mass ratio of 1:1. The suspension was dried at 80 °C for 24 h and afterwards carbonised at 800 °C as described above for the carbonisation process. Afterwards, the activated carbon was washed with distilled water. 

### 2.3. Materials Characterisation

The particle size distributions of the microspheres were measured using a Mastersizer 3000 laser diffraction particle analyser (Malvern), and the data were analysed using the Mie model [26]. Raman measurements to characterise the microspheres were performed using a MultiRam (Bruker Optik GmbH, Ettlingen, Germany) with a laser power of 100 mW and a wavelength of 1064 nm. To investigate the morphology, SEM images of the microspheres were recorded with a FEI Quanta FEG 650 microscope at an accelerating voltage of 5 kV using a SE detector. FIB/SEM measurements were carried out at a Scios LoVac Dual Beam FIB/SEM (FEI/ThermoFisher, Waltham, MA, USA) device at an acceleration voltage of 5 kV. The porosity of the beads was determined by investigations on the sedimentation volume and the packing density using a Universal 320 R centrifuge (Hettich, Kirchlengern, Germany). Further, the carbon and nitrogen contents were determined by a Vario EL III (Elementar, Langenselbold, Germany) in accordance with DIN 51721 [27] and the thermal behaviour of the particles was investigated using an STA 449 F5 Jupiter (Netzsch, Selb, Germany) analysing system under Ar atmosphere with a heating rate of 5 K min^−1^ up to 1200 °C.

The specific surface area, pore size distribution and pore volume of precursors and carbons were determined by nitrogen physisorption experiments using a Quadrasorb SI (Quantachrome Instruments,) at 77 K. Prior to the measurements, the samples were outgassed in a FloVac degasser for 24 h in a dynamic vacuum at 70 °C and 150 °C for cellulosic materials (about 200 mg) and carbons (about 25 mg), respectively. The specific surface area was calculated using the Brunauer-Emmett-Teller (BET) method at relative pressures of p/p_0_ = 0.1–0.3 [28]. The t-plot method was used to calculate the micropore surface area [29]. The total pore volumes were calculated using the Gurvich rule at a relative pressure of p/p_0_ = 0.9, while the pore size distribution was evaluated using the quenched solid density functional theory (QSDFT) for slit and cylindrical pores of carbons [30,31,32]. For the calculations, the software Quadrawin 5.11 (Quantachrome Instruments) was used. Furthermore, the graphitic and disordered parts of the carbons were investigated using a DXR SmartRaman (Thermo Scientific) spectrometer with a wavelength of 532 nm and a laser power of 9 mW; the spectra were analysed using Lorentzian fitting models. SEM images of the carbons were recorded using an LEO Gemini 1530 (Zeiss, Jena, Germany) at an accelerating voltage of 20 kV. An SE2 detector was used. Powder X-ray diffraction (XRD) experiments were carried out in transmission mode on a STOE STADI P powder diffractometer with a curved Ge(111) crystal monochromator using a Dectris 1 K detector. The samples were tested with Cu Kα_1_ radiation of 1.54056 Å wavelength, a scan range of 5° < 2θ < 100°, and a step size of Δ2θ = 0.01°. For the preparation, powder samples were glued as a thin layer between acetate films. X-ray photoelectron spectroscopy (XPS) measurements were performed using a PHI 5600 CI (Physical Electronics, Chanhassen, MN, USA) spectrometer. A non-monochromated Al Kα radiation (200 W) was used at a pass energy of 29.35 eV.

### 2.4. Electrochemical Measurements

The electrodes for the electrochemical measurements were prepared from a mixture of 95% carbon material and 5% binder solution (PVDF in acetone) and homogenised in an ultrasonic bath. The electrode mass was then dropped onto steel current collectors with a diameter of 12 mm, the mass loading per electrode was about 5 mg. A symmetrical two-electrode configuration in Swagelok cells was used. Whatman glass fibre was used as a separator between the electrodes, and 250 µL of 6 M KOH solution was used as the electrolyte. The measurements were carried out at 25 °C in a climatic chamber using a multichannel potentiostat from BioLogic. Cells were tested by cyclic voltammetry (CV) at scan rates ranging from 2 to 500 mV s^−1^ and by galvanostatic cycling with potential limitations at current rates ranging from 1 to 10 A g^−1^. The potential window for measurements was fixed between 0 and 0.8 V. 

The specific capacitances (*C_spec_*) for the electrodes of the supercapacitor were calculated as follows (1):

(1) $$C_{spec}=\frac{I_{spec}}{\frac{\Delta U}{\Delta t}}$$

*I_spec_* (A g^−1^) is the applied current per mass, and Δ*U*/Δ*t* is the slope of the galvanostatic discharge curve, excluding the iR drop. The energy (*E*, Wh kg^−1^) and power (*P*, W kg^−1^) densities were calculated using Equations (2) and (3):

(2) $$\mathrm{Energy}\;\mathrm{density}:\;E=0.5\times C_{spec}*U2$$

(3) $$\mathrm{Power}\;\mathrm{density}:\;P=\frac{E}{\Delta t}$$

## 3. Results

### 3.1. Cellulose Acetate Butyrate Microspheres

**Figure 3:**
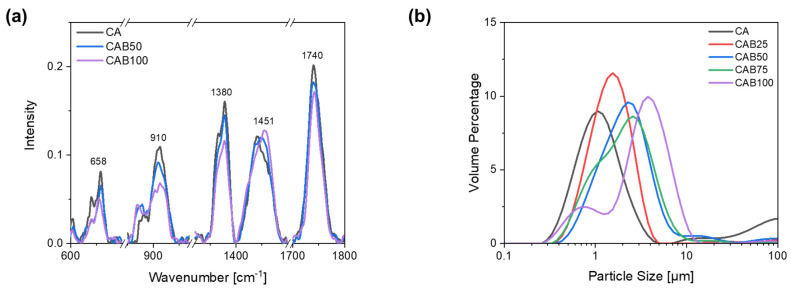
(**a**) Raman spectra for characteristic bands and (**b**) particle size distribution of microspheres with different amounts of CAB.

Figure 3a shows Raman spectra of the microspheres prepared with different amounts of CAB for characteristic bands of cellulose acetate and cellulose acetate butyrate. The complete Raman spectra of the microspheres with different CAB amounts as well as the Raman spectra of the raw materials CA2.5 and CAB powder, for comparison, are shown in Figure S1, Supplementary Material. 

All materials have a strong band at a wavenumber of 1740 cm^−1^, which is typical for esters and is caused by C=O stretching vibrations of the acetyl group. The band at 1380 cm^−1^ is also present in all prepared materials as well as in the raw material, which can be attributed to various deformation vibrations of the cellulose structure as δ(CH2), δ(HCC), δ(HCO), δ(COH). Other bands, which are caused by signals from the cellulose backbone, are in the range of 1200–1000 cm^−1^. The band at a wavenumber of 658 cm^−1^ indicates signals from the acetyl group, in particular deformation vibrations of the C=O bonds in ester groups. Cellulose acetate has a characteristic band at 1437 cm^−1^, which can be attributed to deformation vibrations of the CH_3_ group. In contrast, cellulose acetate butyrate has a characteristic band at 1451 cm^−1^, which is assigned to the amorphous phase. In addition, another typical band is seen for cellulose acetate butyrate at 866 cm^−1^, caused by deformation vibrations of the C-O-O-H and O-C-O bonds in the molecular plane [33,34,35,36].

With the Raman spectra we confirmed that the prepared microspheres contain cellulose acetate butyrate, depending on the amount. The two bands at a wavenumber of 866 cm^−1^ and 1451 cm^−1^, which are typical for cellulose acetate butyrate, show a clear differentiation to the cellulose acetate microspheres. The band at 1451 cm^−1^ is slightly shifted depending on the CAB amount of the microspheres. In contrast, the band at 1740 cm^−1^, which is caused by C=O stretching vibrations of the acetyl group, decreases with an increasing amount of CAB. The intensity of the bands typical for cellulose (1380, 1200–1000, 910, 658 cm^−1^) also decreases with a higher amount of CAB in the microspheres. 

For the CA microspheres, it is known from other studies that it is possible to obtain particle sizes below 5 µm with a narrow particle size distribution [2,23]. As the amount of cellulose acetate butyrate in the microspheres increases, the particle size rises and the particle size distribution becomes wider, as shown in Figure 3b. The particles with 0% CAB (CA) have a median d_50_ value of 1.3 µm, whereas those with 50% and 100% CAB have values of 2.2 µm and 3.4 µm, respectively. The particle size distribution for the microspheres containing 75% CAB shows a shoulder at about 1 µm, indicating the beginning of particle segregation. When the amount of CAB is further increased to 100%, two fractions of different particle sizes are recognisable. There are very small particles with an average diameter of 0.8 µm and larger particles with an average diameter of 4.2 µm. 

**Figure 4:**
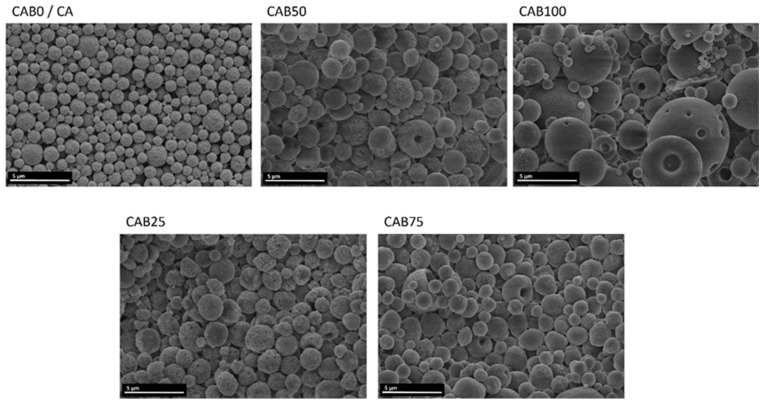
SEM images of the microspheres with various CAB amounts.

The different particle sizes as well as the morphology of the microspheres and their surface structure were investigated by scanning electron microscopy, shown in Figure 4. The CA microspheres without CAB are relatively homogeneous in terms of particle size and structure, in agreement with other studies [2,23]. The microspheres have a netlike structure at the surface and appear to be highly porous. By adding CAB solution in the preparation process of the microspheres, the particle sizes become larger and the distribution in the particle size significantly wider, in accordance with the particle size measurements. In addition, the two fractions of different particle sizes for CAB100 with the very small and large particles are depicted. 

The SEM images of the particles with different amounts of CAB show that for all amounts spherical particles are created, but the structure of the microspheres changes (Figure 4). As the amount of CAB increases, the surface of the microspheres becomes smoother and the microspheres appear to be “filled”, instead of the porous structure with a netlike surface of the CA microspheres. For some of the microspheres, which seem to be filled and have a relatively smooth surface, dents appear at the surface. These holes might arise through an increase in internal pressure within the microspheres, caused by the filling. The microspheres CAB25 and CAB50 show two different structures and sometimes both structures coexist together on the same particle. One half is rather porous and has a netlike structure, while the other half has a smooth surface. The differences in the formation of the microspheres between cellulose acetate and cellulose acetate butyrate are very evident in these microspheres, which show both structures simultaneously. Accordingly, CAB25, CAB50 and CAB75, which were prepared from a mixture of the two solutions (CA and CAB), contain individual microspheres of CA and CAB, as well as mixed microspheres, depending on the amount of CAB. 

**Figure 5:**
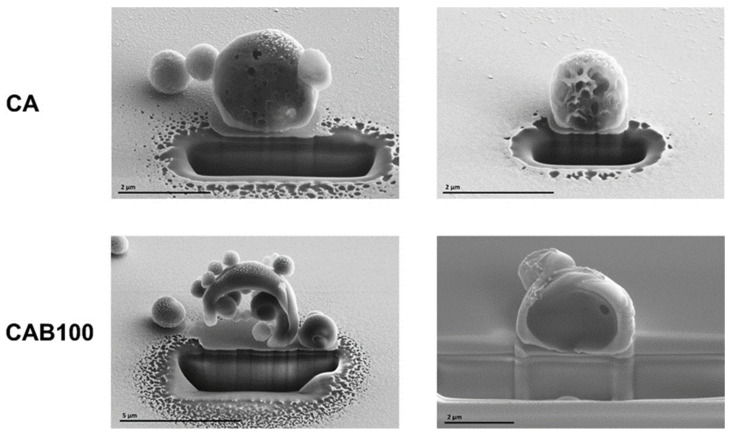
SEM images of CA and CAB100 microspheres sliced using a Focused Ion Beam (FIB).

SEM measurements of Focused Ion Beam (FIB)-sliced samples enabled for the first time analysis of the inner structure of the microspheres (Figure 5). The CA microspheres have, as shown before by the SEM images, a porous and netlike structure on the surface, which also extends into the inner part of the microspheres. This results in a high porosity of the CA microspheres. The inner structure of the CAB100 microspheres differs significantly from that of CA. For the cellulose acetate butyrate microspheres, two types can be detected in relation to the inner structure. In some cases, it was found that several smaller microspheres are enclosed within a larger microsphere, while in others the microspheres are almost completely filled. Up to now, it was not possible to clarify when the formation of the microspheres leads to a complete filling and when it leads to the inclusion of smaller microspheres. Furthermore, it is not clear what the proportion of each type of the microspheres (filled or enclosed microspheres) is. Possible influencing factors are the choice of surfactant and the temperature during the preparation process. However, a number of further investigations are required to clarify this phenomenon and to identify the key components influencing the process. The different inner structures of the microspheres have a major impact on the subsequent formation of the carbons in terms of the specific surface area and pore structure. 

The porosity of the cellulose acetate (butyrate) microspheres was determined from the sedimentation volume and the packing density of the beads according to the method of Thümmler et al. [2]. The microspheres were dispersed in water and then centrifuged at 4500 rpm for 30 min. Afterwards, the sedimentation volume *V_Sed_* is calculated from the difference between the total volume *V_tot_* and the volume of the water supernatant *V_WS_*, *w_CA_* is the solid content of the microspheres. For the calculation it is assumed that the density of the microspheres is *ρ* = 1.25 g cm^−3^. Further, the packing density PD (4) and the porosity *ε* (5) are calculated as follows:

(4) $$PD=10*\frac{w_{CA}*V_{tot}}{V_{Sed}}$$

(5) $$\varepsilon =100*(1-\frac{4}{3}*\frac{PD}{\rho })$$

**Figure 6:**
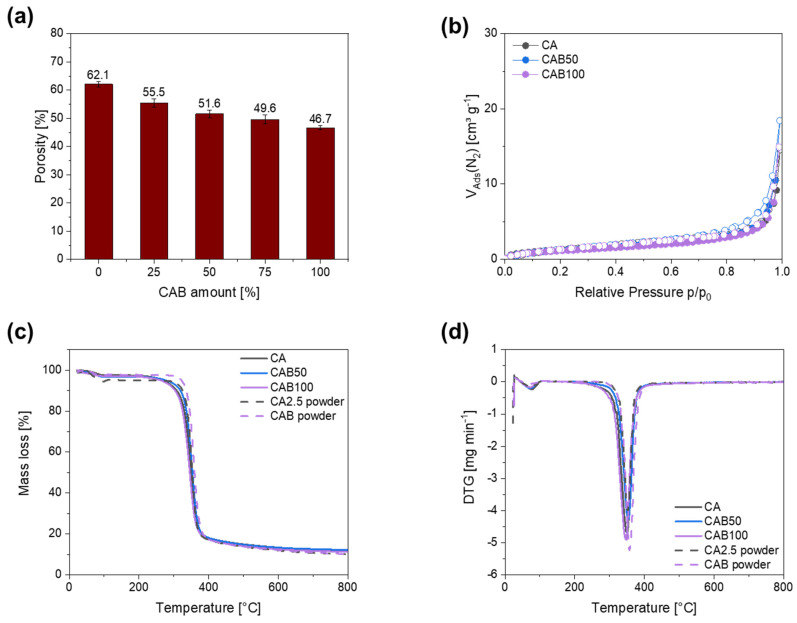
(**a**) Porosity depending on the CAB amount, (**b**) nitrogen physisorption isotherms, (**c**) mass loss and (**d**) DTG for several microspheres.

As shown in Table 1 and Figure 6a, the porosity of the microspheres decreases with a higher amount of cellulose acetate butyrate. While pure cellulose acetate microspheres have a porosity of 62.1%, the porosity drops to 55.5% with the addition of 25% CAB. Afterwards, the porosity decreases a bit more slightly and the CAB100 microspheres have a porosity of only 46.7%. The large differences between the CAB microspheres and the CA microspheres are probably due to the significantly larger particle sizes of the microspheres and, therefore, a smaller void space between the microspheres. Furthermore, the SEM images show that the porous structure on the surface of CA microspheres changes to a smoother surface with an increasing amount of CAB. With a smoother surface, water adsorption in the pores probably decreases and, therefore, the “inner” porosity of the microspheres becomes smaller. These results are also consistent with the FIB/SEM measurements, which provide information on the inner structure of the microspheres. While the CA microspheres have a relatively porous structure, the CAB100 microspheres are either completely filled or contain several small microspheres. With the porosity determined by measuring the difference between the total volume and the sedimentation volume, the total empty space within and between the microspheres is measured and it contains no reliable information about the pore volume and the pore size distribution of the microspheres [3].

For the further preparation of carbons, the amount of carbon in the cellulose acetate (butyrate) microspheres is an important parameter. The carbon content in the raw (purchased) materials CA2.5 and CAB is 48.0% and 51.8%, respectively. The microspheres from cellulose acetate have a slightly higher carbon content of 49.4% compared to the raw material, probably due to the preparation process of the microspheres, such as the use of methylcellulose. With the addition of cellulose acetate butyrate in the preparation process, the amount of carbon in the microspheres increases slightly and the carbon content of CAB100 (51.3%) is very similar to the raw material of cellulose acetate butyrate. 

Nitrogen physisorption measurements were carried out to obtain information about the specific surface area. The nitrogen physisorption isotherms as well as their hysteresis forms are assigned according to IUPAC [37]. The nitrogen physisorption isotherms for the prepared microspheres (Figure 6b and Figure S2) are very similar and can be assigned to type III without hysteresis. Characteristic for type III is that there is no identifiable monolayer and the interactions between the adsorbents and adsorptive are very weak. The specific surface area does not differ significantly, ranging from 4.4 to 4.9 m^2^ g^−1^ for CA, CAB50 and CAB100. Therefore, the different particle sizes, morphologies and porosities of the microspheres have no influence on their specific surface area. 

Furthermore, the decomposition behaviour of the microspheres and the raw materials was investigated using thermogravimetric analysis (Figure 6c,d). The decomposition temperature for the microspheres varies between 320 and 330 °C and is slightly higher for the raw materials CA2.5 and CAB powder with 336 and 340 °C, respectively. For all samples, the residual mass at a temperature of 800 °C is 14.5–15% with a slightly increased value for a higher amount of CAB in the samples. As the decomposition process is very similar, the same carbonisation conditions are used for CAB and CA microspheres. 

### 3.2. Carbons from the Prepared Microspheres

An important method for characterising the carbons is the nitrogen physisorption, also used in the previous part to characterise the microspheres. In addition to the specific surface area, it also provides information about pore structure, pore volume and the microporosity of the carbons (Table 2). 

**Figure 7:**
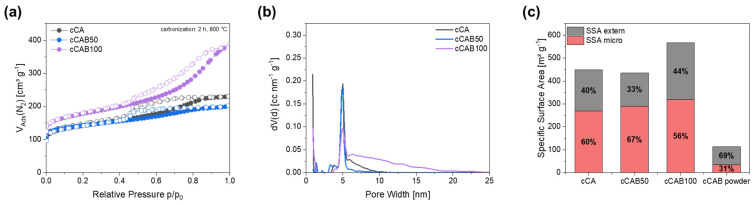
(**a**) Nitrogen physisorption isotherms with (**b**) corresponding pore size distribution and (**c**) classification into extern and micro specific surface area of carbons from microspheres with different amounts of CAB as well as carbonised CAB powder.

The specific surface area of the carbons changes only slightly for microspheres containing a mixture of cellulose acetate and cellulose acetate butyrate (CAB50) compared to carbons from cellulose acetate microspheres, with values of 448.6 m^2^ g^−1^ for cCA and 435.0 m^2^ g^−1^ for cCAB50. However, it is interesting to note that the proportion of the specific surface area due to micropores is significantly higher for cCAB50 (67%) than for cCA (60%) (Figure 7c). In contrast, the specific surface area of the carbons prepared from microspheres entirely from cellulose acetate butyrate (CAB100) is significantly higher, with a value of 566.7 m^2^ g^−1^. The higher surface area of the carbons from cellulose acetate butyrate microspheres compared to cellulose acetate microspheres is probably related to their inner structure. As shown before, some of the larger cellulose acetate butyrate microspheres additionally contain small microspheres which are released during the decomposition to carbon, thus providing additional surface area. The filled microspheres might also lead to an increased surface area, since the structure is destroyed during carbonisation. The proportion of the specific surface area related to micropores decreases again to 56.2%. For comparison, cellulose acetate butyrate powder was carbonised under the same conditions. The specific surface area of this carbon of 114.0 m^2^ g^−1^ is significantly lower in contrast to carbon prepared from the spherical particles of CAB100. Additionally, the proportion of micropores contributing to the specific surface area is only 31.4%. 

The sorption isotherms of all carbons without activation (Figure 7a) show a type IV shape, whereby the type of hysteresis depends on the amount of CAB. For cCA and cCAB50, the hysteresis has a type 2 form, whereas cCAB100 shows a type 4 shape. Characteristic for the type IV isotherm is the occurrence of a turning point located at low relative pressure (p/p_0_ < 0.1), which is related to the filling of the first monolayer. Type IV is typical for mesoporous materials, and after the filling of the first monolayer pore condensation takes place. At a relative pressure of p/p_0_ > 0.9, a saturation plateau is reached, whereby this plateau becomes smaller as the amount of CAB increases. In addition, the slope of the isotherm for cCAB100 is significantly steeper in the range of 0.4 < p/p_0_ < 0.9, indicating a larger volume of mesopores filled in this relative pressure range. 

The hysteresis type contains further information about the structure of the carbon, but both the type 2 and type 4 shapes assigned to the carbons from the respective microspheres imply complex pore structures. Type 2 is typical for a variety of network effects such as pore blocking and ink bottle-shaped pores, whereas type 4 has a complex network containing micropores and mesopores [31]. This is also confirmed by the pore size distribution in Figure 7b, where all carbons show a mixture of micro- and mesopores with a peak < 1 nm and a peak around 5 nm. The proportion of mesopores with a pore diameter > 5 nm increases significantly with a higher CAB content, resulting in a higher mean pore diameter of 6.7 nm for cCAB100. In contrast, cCA and cCAB50 have smaller mean pore diameters of 3.9 nm and 4.1 nm.

**Figure 8:**
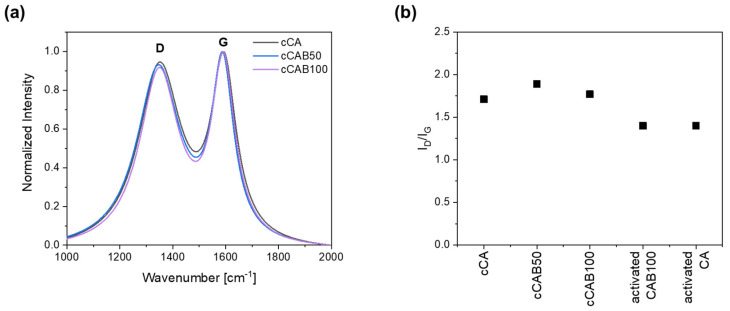
(**a**) Raman spectra and (**b**) I_D_/I_G_ ratio for non-activated and activated carbons from microspheres with different CAB amounts.

Raman spectra of the prepared carbons from the microspheres show intense D and G bands, pointing to the existence of both graphitic and disordered regions (Figure 8a). The D band, located at 1350–1355 cm^−1^, arises from the disordered parts, whereas the G band, located at 1580–1600 cm^−1^, arises from the graphitic parts. The intensity ratio I_D_/I_G_ between the both integrated peaks is used to obtain information about the degree of graphitisation. As the intensity of the D band increases, the proportion of disordered carbon parts gets higher and the crystallite size of graphite decreases [38,39].

The degree of graphitisation initially decreases slightly with the introduction of CAB components with an I_D_/I_G_ value of 1.7 for cCA to 1.9 for cCAB50 (Figure 8b). With a further increase in the CAB amount (cCAB100), the I_D_/I_G_ value rises again to 1.9. Overall, however, the values are within a very narrow range of 1.7 to 1.9, so that no clear effect can be established by changing the cellulose acetate butyrate content on the degree of graphitisation. With the activation of the carbons, the degree of graphitisation increases, giving the I_D_/I_G_ value of 1.4 for both the activated CA and CAB100. 

**Figure 9:**
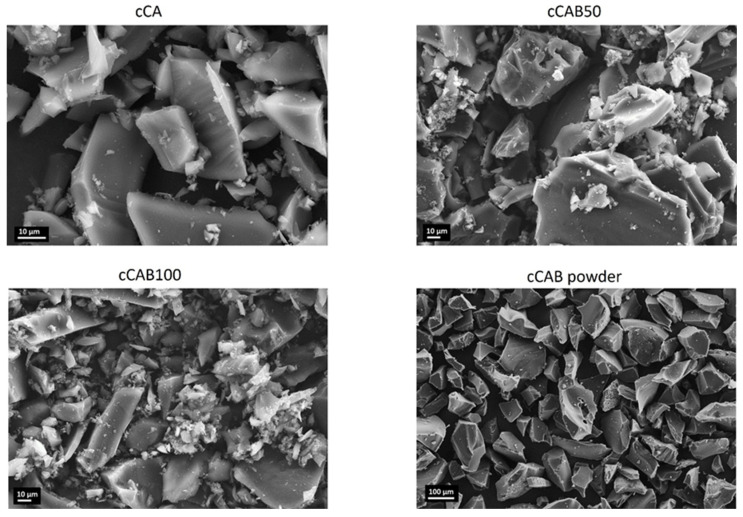
SEM images of carbons from microspheres with different CAB amounts as well as from CAB powder.

The SEM images in Figure 9 show the morphology of carbon particles prepared from the microspheres with different amounts of CAB as well as the carbon from the raw material. In general, all the carbons have a relatively smooth surface, although the particle sizes vary considerably. With a higher amount of CAB, the particles generally become a bit smaller. It is also noticeable that the proportion of very small carbon particles increases and thus the particle size distribution becomes wider. The higher number of these very small particles is probably responsible for the higher specific surface area of cCAB100 compared to cCA. The carbon from the CAB powder has a very smooth surface and comparatively large particles of about 100 µm. These results in a much lower specific surface area and a smaller pore volume compared to the carbons from the microspheres. 

**Figure 10:**
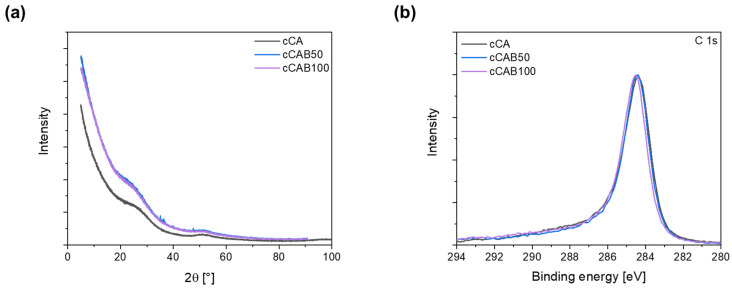
(**a**) X-ray diffraction patterns (Cu Kα_1_ radiation, λ = 1.54056 Å) and (**b**) XPS spectra for carbons from microspheres with different CAB amounts.

Irrespective of the CAB amount of the microspheres, none of the carbons show any sharp reflections in the X-ray diffraction pattern (Figure 10a), which would indicate crystalline phases. The broad reflections at about 23° and 50° can be assigned to the (002) and (100) planes, which are typical for graphitic and amorphous carbons [40,41]. XPS measurements show that the elemental concentrations of C and O at the surface depend on the CAB content of the carbons (Table 3). The carbon concentration increases from 90.0% for cCA to 95.6% for cCAB100, while the oxygen concentration decreases with an increasing CAB content of the carbons. The C 1s spectra in Figure 10b are relatively similar and do not show any clear differences with regard to the distribution of functional groups on the surface of the carbons. All carbons show a distinct peak at a binding energy of 284.8 eV, which can be attributed to the carbon bonds C=C, C-C and C-H. It can also be assumed that each carbon has a small proportion of bonds with oxygen in the form of alcohols (286–287 eV) and carbonates (289–291 eV) [42].

**Figure 11:**
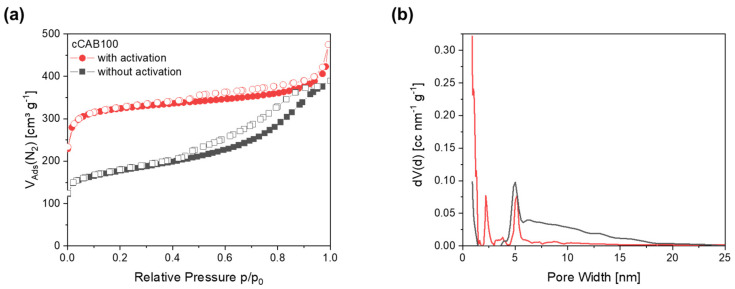
(**a**) Nitrogen physisorption isotherms and (**b**) corresponding pore size distributions for activated and non-activated carbons prepared from CAB100 microspheres.

In contrast to the non-activated carbons, the activated carbon derived from CAB100 has a type I-shaped isotherm with a rapid increase at low pressures p/p_0_ < 0.1, caused by the filling of the micropores (Figure 11a). In general, type I isotherms are typical for microporous materials. Furthermore, a minimal hysteresis of type 4 can be observed for the activated carbon, indicating a small proportion of mesopores. Activation with KOH, therefore, results in a higher specific surface area of 948.7 m^2^ g^−1^ and in a change from a more mesoporous to a microporous structure, which is also reflected in the pore size distribution as well as in a higher proportion of the specific surface area caused by micropores (86%). The pore size distribution of the non-activated carbon shows a high proportion of mesopores with a peak at 5 nm (Figure 11b). After activation, the proportion of micropores < 1 nm is generally slightly larger and the proportion of mesopores decreases. Furthermore, an additional peak at 2.2 nm occurs during activation. This results in a smaller average pore diameter of the activated CAB100 carbon of 3.0 nm in contrast to the non-activated carbon with a pore diameter of 6.7 nm. The combination of numerous micropores with smaller mesopores appears to be advantageous for the accessibility of the electrolyte ions for subsequent use as electrode material in supercapacitors. The specific surface area of the activated carbon derived from cellulose acetate microspheres is slightly higher and the average pore diameter is smaller than that of the activated CAB100. A detailed description of the characteristics of the activated CA carbon is given in further studies [23]. The larger specific surface area of activated carbons from CA microspheres compared to CAB100 microspheres could be due to a more effective activation of the material with KOH due to the higher porosity of the CA microspheres. The SEM images of the activated carbons derived from CAB100 microspheres in Figure S3 also show a significantly altered surface structure compared to the non-activated carbons. The surface of the individual particles is attacked by the activation with KOH and many small particles and increased pores are formed resulting in an increased specific surface area. 

### 3.3. Electrochemical Measurements

**Figure 12:**
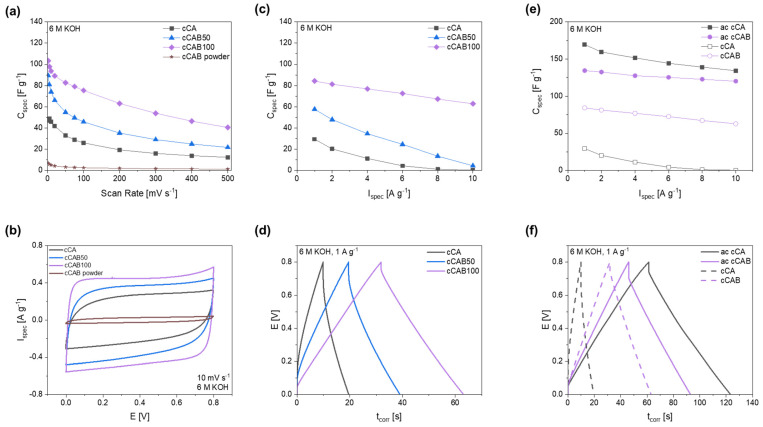
(**a**) Specific capacitances depending on the scan rate with (**b**) corresponding cyclic voltammograms at a scan rate of 10 mV s^−1^, (**c**) specific capacitances depending on the current rate with (**d**) corresponding GCPL curves at 1 A g^−1^ for carbons prepared from microspheres with different CAB amounts, as well as (**e**) specific capacitances of activated and non-activated carbons from CA and CAB100 microspheres with (**f**) corresponding GCPL curves at 1 A g^−1^.

By adding cellulose acetate butyrate during the preparation of the microspheres, carbons can be produced which achieve higher specific capacitances compared to carbons from CA microspheres (Figure 12a). The electrochemical performance depends on the amount of CAB used. The higher the amount of CAB in the microspheres used as precursors, the higher the specific capacitances that can be achieved in both cyclic voltammetry and GCPL tests. Thus, at a scan rate of 10 mV s^−1^, the capacitance can be increased from 46.0 F g^−1^ for cCA to 74.1 F g^−1^ at a CAB amount of 50% (cCAB50) and further to 93.6 F g^−1^ for cCAB100. In contrast, the carbon from the CAB raw material (cCAB powder) leads to very low capacities of 5.1 F g^−1^ at a scan rate of 10 mV s^−1^. Therefore, the conversion of CAB or CA precursors into the spherical form is crucial for the suitability of the resulting carbons as electrode materials for electrochemical applications. The cyclic voltammogram at a scan rate of 10 mV s^−1^ in Figure 12b shows an almost ideal rectangular shape for cCAB100, which deviates only slightly for lower amounts of CAB in the carbons. The rectangular shape is typical for an ideal electrochemical double layer capacitor, which has no redox reaction or any noticeable resistance within the cell. For carbons from CAB powder, the curve is rather slit-like, indicating high internal resistances in the cell. However, at higher scan rates, the capacitances drop for all materials and reach values between 12 and 40 F g^−1^ at a fast scan rate of 500 mV s^−1^ (charge/discharge time 3.2 s). 

The results of the CV measurements with higher capacitances for carbons, prepared from precursors with a higher CAB amount, can also be confirmed by GCPL tests (Figure 12c). At a current rate of 1 A g^−1^, a comparatively high specific capacitance of 84.2 F g^−1^ is achieved for cCAB100, while cCA shows a value of only 29.4 F g^−1^. In addition, cCAB100 shows good stability at higher current densities compared to the carbons produced from precursors with a lower amount of CAB with a specific capacitance of 62.8 F g^−1^ at 10 A g^−1^. The GCPL curves at a current density of 1 A g^−1^ in Figure 12d show a triangular shape with a small iR drop for cCAB100, whereas the shape deviates slightly with lower amounts of CAB and the iR drop, which indicates inner resistances in the cell, becomes larger. 

The charging/discharging at a current rate of 1 A g^−1^ requires about 63 s for cCAB100 and is, therefore, more than twice as fast as the CV measurements at a scan rate of 10 mV s^−1^ with a charging/discharging time of about 160 s. 

Promising specific capacitances have already been achieved with carbon from cellulose acetate butyrate microspheres without activating the material. For further improving the performance for the use as electrode material in supercapacitors, the CAB100 microspheres were activated with KOH in a mass ratio of 1:1. However, in contrast to the non-activated carbons, the activated carbons from CAB100 microspheres achieve lower specific capacitances in the CGPL measurements in Figure 12e compared to the activated carbons from CA microspheres. At a current rate of 1 A g^−1^, the activated CAB100 has a specific capacitance of 134.4 F g^−1^, whereas the activated CA achieves 169.5 F g^−1^. The activated carbons of CA and CAB100 differ primarily in their specific surface area, which is, at 1197.6 m^2^ g^−1^, significantly larger for CA than for CAB100, with a value of 948.7 m^2^ g^−1^. Both surfaces are mostly caused by micropores (about 85%). The larger specific surface area of activated CA is probably the reason for the higher specific capacitance compared to CAB100. Also, the iR drop in the GCPL curve at 1 A g^−1^ in Figure 12f is significantly larger for activated CAB100, indicating possible resistances within the cell influencing the specific capacitance and the electrochemical stability. 

**Figure 13:**
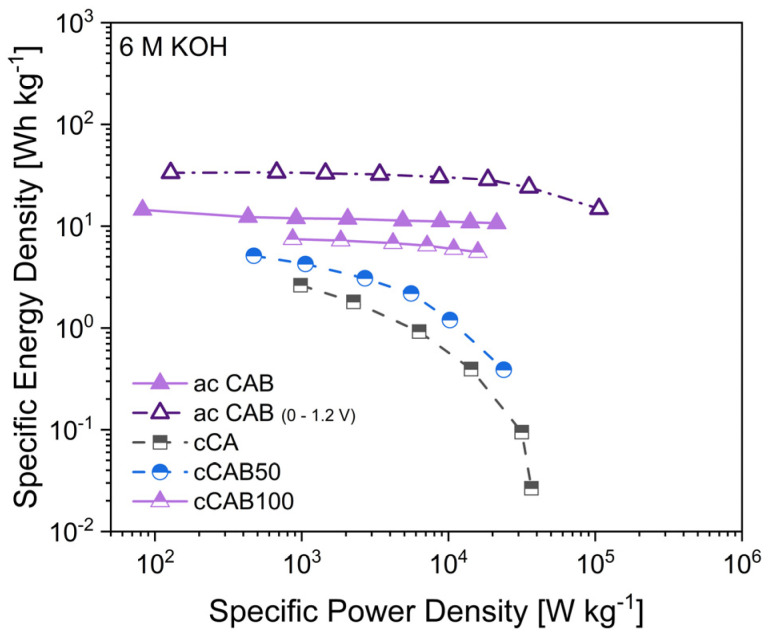
Ragone plot of supercapacitors with carbon electrodes derived from microspheres with different amount of CAB.

The specific energy and power densities, shown in the Ragone plot in Figure 13, are an important characteristic for comparing the performance of supercapacitors. The higher the amount of CAB of the microspheres used as precursor for the carbons, the higher the energy density when used as electrode material. At 1 A g^−1^, the energy densities for cCA, cCAB50 and cCAB100 are 2.6, 5.1 and 7.5 Wh kg^−1^, respectively. While the energy density for cCA and cCAB50 decreases sharply at higher current rates, it remains relatively stable for cCAB100. A higher proportion of CAB therefore contributes to stability and better performance.

In order to achieve higher specific capacitances and thus higher energy densities, activated carbons from microspheres of CA and CAB were used. The energy densities of the supercapacitors with activated carbons are 12 Wh kg^−1^ for CAB microspheres and 15 Wh kg^−1^ for CA microspheres with comparable power densities of 0.9 kW kg^−1^ at a current density of 1 A g^−1^ in a potential window of 0–0.8 V. By extending the potential window during cycling from 0–0.8 V to 0–1.2 V, both the energy and power densities of the supercapacitor can be further optimised. At 1 A g^−1^, the energy density reaches 33 Wh kg^−1^ at a power density of 1.5 kW kg^−1^. However, a prerequisite for extending the potential range is the stability of the electrolyte, which for aqueous electrolytes is limited by the decomposition of water at a potential > 1.23 V [43]. In addition, a good long-term stability over 2000 cycles can be shown for electrodes with activated carbon from CAB microspheres when cycled in a potential window of 0–1.2 V (Figure S4, Supplementary Material).

## 4. Conclusions

In conclusion, the acetate method represents a viable approach for obtaining microspheres with a particle size of less than 5 µm, even when cellulose acetate butyrate instead of cellulose acetate is used as a precursor. The substitution of cellulose acetate with cellulose acetate butyrate during the preparation process significantly changes the structure and properties of the microspheres. This substitution results in smoother surfaces with some dents and a less porous inner structure, leading to reduced porosity and increased particle size with a broader particle size distribution as the amount of CAB in the microspheres increases. After carbonisation, the microspheres derived from CAB have a notably higher specific surface area of 567 m^2^ g^−1^ compared to those derived from CA with 449 m^2^ g^−1^, indicating the potential for improved electrochemical performance. The activation of the cellulose acetate butyrate microspheres with KOH can further increase the specific surface area and change the pore structure of the carbons, resulting in a higher proportion of micropores. These prepared carbons show promising results as electrode materials in symmetrical supercapacitors with 6 M KOH aqueous electrolytes. Carbons derived from CAB100 exhibit a specific capacitance of 84.2 F g^−1^ at 1 A g^−1^ and show stable performance at higher current densities with 62.8 F g^−1^ at 10 A g^−1^, too. Activation with KOH further enhances the performance leading to a specific capacitance of 134.4 F g^−1^ at a current density of 1 A g^−1^. In addition, the conversion of cellulose-derivative precursors into a spherical structure positively effects the structure of the carbons and, therefore, their electrochemical performance. Carbons derived solely from CAB powder exhibit a lower specific surface area of 114 m^2^ g^−1^ and inferior electrochemical performance when used as electrode materials.

Overall, these results highlight the potential of carbons from CAB-derived microspheres as promising candidates for high performance supercapacitor electrodes, especially when optimised by activation processes. Furthermore, the preparation of spherical microspheres with cellulose acetate butyrate can serve as a basis for the preparation of microspheres with other cellulose derivatives. The changed properties and surface functionalities compared to the cellulose microspheres may also have advantages for other applications besides electrochemical energy storage. 

## Figures and Tables

**Table 1:** Characteristics of the prepared microspheres.

CAB Amount [%]	Average Particle Size [µm]	Porosity [%]	Specific Surface Area [m^2^ g^−1^]	C Amount [%]	Decomposition Temperature [°C]
0	1.3	62.1	4.9	49.4	327.4
25	1.5	55.5	6.4	49.6	-
50	2.2	51.6	4.4	50.8	333.2
75	2.2	49.6	3.6	52.0	-
100	3.4	46.7	4.6	51.3	320.9
CA2.5 powder	-	-	15.4	48.0	336.0
CAB powder	-	-	3.7	51.8	340.3

**Table 2:** Characteristics of non-activated (c) and activated (ac) carbons from CA and CAB microspheres compared to carbons from the CAB raw material.

	Specific Surface Area [m^2^ g^−1^]	S_micro_ [m^2^ g^−1^]	Average Pore Width [nm]	Pore Volume [cm³ g^−1^]	Pore Volume Micro [cm³ g^−1^]	I_D_/I_G_
cCA	448.6	268.8 (60%)	3.9	0.34	0.15 (43%)	1.7
cCAB50	435.0	290.7 (67%)	4.1	0.30	0.16 (53%)	1.9
cCAB100	566.7	318.4 (56%)	6.7	0.54	0.17 (32%)	1.7
cCAB powder	114.0	35.7 (31%)	-	-	-	1.9
ac CAB100	948.7	813.1 (86%)	3.0	0.58	0.44 (76%)	1.4
ac CA	1197.6	1015.6 (85%)	2.1	0.69	0.55 (80%)	1.4

**Table 3:** Atomic concentrations of carbon and oxygen on the surface for carbons obtained from microspheres with different CAB amounts.

	C 1s [%]	O 1s [%]
**cCA**	90.0	10.0
**cCAB50**	92.3	7.7
**cCAB100**	95.6	4.4

## Data Availability

The original contributions presented in the study are included in the article/supplementary material; further inquiries can be directed to the corresponding authors.

## Supplementary Materials

The following supporting information can be downloaded at https://www.mdpi.com/article/10.3390/polym16152176/s1, Figure S1: Raman spectroscopy of (a) microspheres with different amounts of CAB and (b) the raw materials CA2.5 powder and CAB powder, Figure S2: nitrogen physisorption isotherms of CAB25 and CAB75, Figure S3: SEM images of activated carbon derived from CAB100 microspheres, Figure S4: long-term electrochemical performance for activated carbons from CAB microspheres at 1 A g^−1^ for 2000 cycles using 6 M KOH electrolyte.
