# Supplementary material for: Synthesis of Cellulose Acetate Butyrate Microspheres as Precursor for Hard Carbon-Based Electrodes in Symmetric Supercapacitors
Source: Polymers (Basel). 2024 Jul 30;16(15):2176. doi: 10.3390/polym16152176 (PMC11314155; doi:10.3390/polym16152176)
Supplement: Supplementary file 1 [file polymers-16-02176-s001.zip › polymers-3124044-supplementary.pdf]

Synthesis of cellulose acetate butyrate microspheres as precursor for hard carbon-based electrodes in symmetric supercapacitors

Johanna Fischer [1,3,*], Katrin Thümmler [1], Igor Zlotnikov [2], Daria Mikhailova [3], Steffen Fischer [1,*]

1 TUD Dresden University of Technology, Institute of plant and wood chemistry, Pienner Str. 19, 01737 Tharandt, Germany; Johanna.fischer1@tu-dresden.de (J.F.); Katrin.thuemmler@tu-dresden.de (K.T.); steffen.fischer@tu-dresden.de (S.F.)
2 TUD Dresden University of Technology, B CUBE – Center for Molecular Bioengineering, Tatzberg 41, 01307 Dresden, Germany; igor.zlotnikov@tu-dresden.de
3 Leibniz Institute for Solid State and Material Research (IFW) Dresden e.V., Institute for Materials Chemistry (IMC), Helmholtzstraße 20, 01069 Dresden, Germany; d.mikhailova@ifw-dresden.de
* Correspondence: Johanna.fischer1@tu-dresden.de (J.F.), steffen.fischer@tu-dresden.de (S.F.)

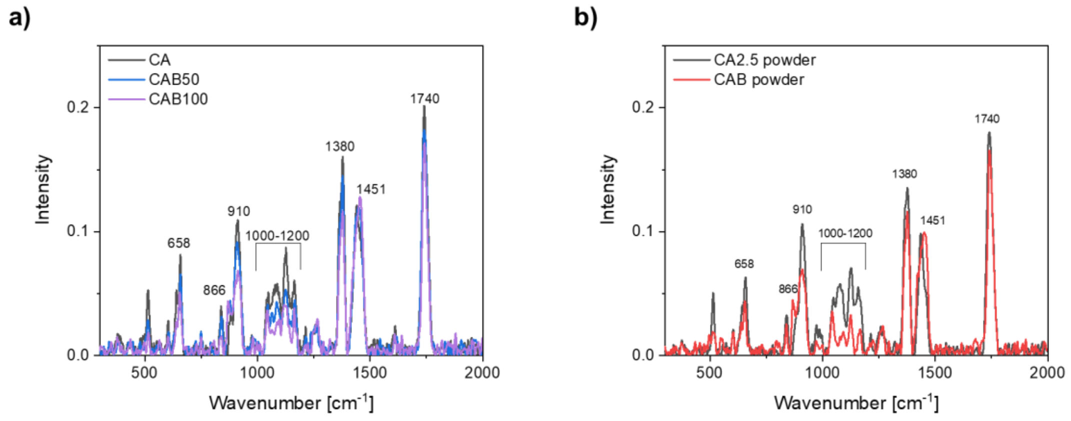

Figure S1. Raman spectroscopy of a) microspheres with different amounts of CAB and b) the raw materials CA2.5 powder and CAB powder.

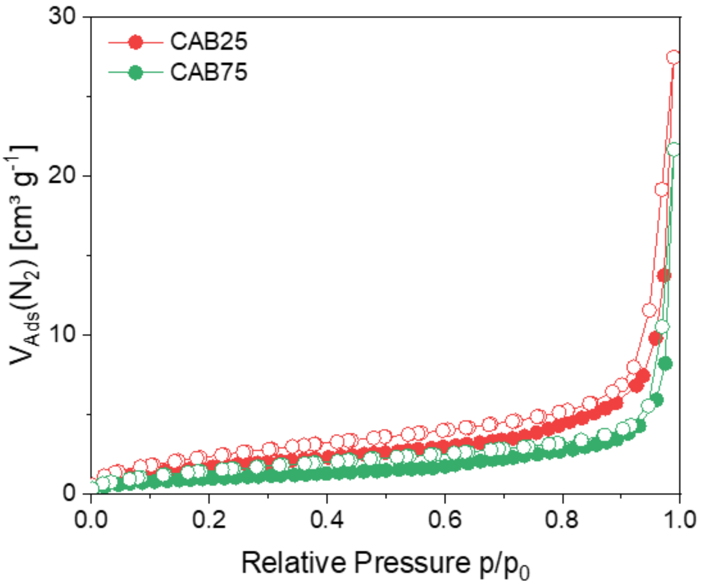

Figure S2. Nitrogen physisorption isotherms of CAB25 and CAB75.

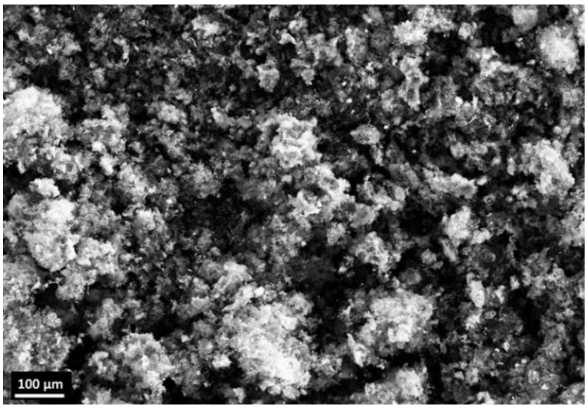

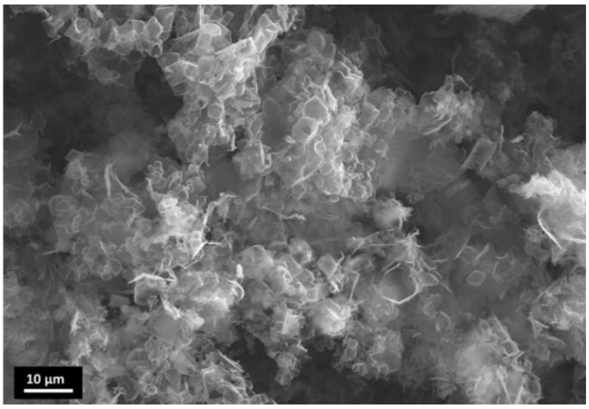

Figure S3. SEM images of activated carbon derived from CAB100 microspheres.

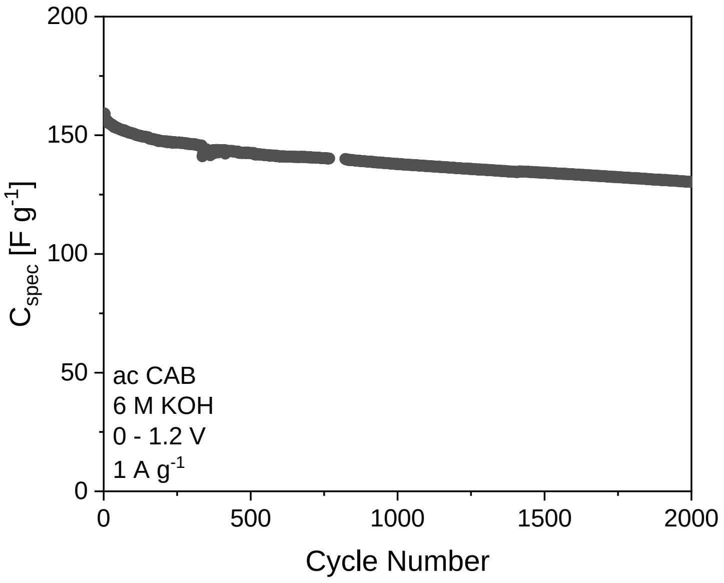

Figure S4. Long-term electrochemical performance for activated carbons from CAB microspheres at 1 A g^{-1} for 2000 cycles using 6 M KOH electrolyte.
